# Effects of exercise training on obesity‐related parameters in people with intellectual disabilities: systematic review and meta‐analysis

**DOI:** 10.1111/jir.12928

**Published:** 2022-03-16

**Authors:** J. Salse‐Batán, M. A. Sanchez‐Lastra, D. Suárez‐Iglesias, C. Ayán Pérez

**Affiliations:** ^1^ Institut Nacional d'Educació Física de Catalunya (INEFC) Universitat de Barcelona (UB) Barcelona Spain; ^2^ Grupo de Investigación Wellness and Movement, Departamento de Didácticas Especiais, Facultade de Ciencias da Educación e do Deporte Universidade de Vigo Pontevedra Spain; ^3^ Instituto de Investigación Sanitaria Galicia Sur (IIS Galicia Sur) Sergas‐UVIGO Vigo Spain; ^4^ VALFIS Research Group, Institute of Biomedicine (IBIOMED), Faculty of Physical Activity and Sports Sciences University of León León Spain

**Keywords:** adiposity, body mass index, exercise, fat percentage, intellectual disability, obesity

## Abstract

**Background:**

Efforts to synthesise existing knowledge concerning the effects of exercise interventions on obesity (i.e. changes in body weight and composition) have been made, but scientific evidence in this matter is still limited. This systematic review and meta‐analysis aims to identify and critically analyse the best available evidence regarding the use of physical exercise as a strategy to attenuate obesity through its effects on adiposity‐related anthropometric parameters in people with intellectual disability (ID).

**Methods:**

Following the Preferred Reporting Items for Systematic Reviews and Meta‐Analyses guidelines, a literature search was performed using PubMed, Scopus, SPORTDiscus, CINAHL and the Cochrane Library through specific keywords up to July 2020. The search adhered to the population, intervention, comparison and outcome strategy. Randomised controlled trials addressing the effects of the exercise intervention on adiposity‐related anthropometric parameters (body mass index, waist circumference, waist–hip ratio, fat percentage or body weight) in children, adolescents and adults with ID were included. The methodological quality of the studies found was evaluated through the PEDro scale.

**Results:**

A total of nine investigations with children and/or adolescents (10–19 years) and 10 investigations with adults (18–70 years) were selected, mostly experiencing mild and moderate ID. Methodological quality was fair in 13 of these publications, good in five and excellent in one. Seventeen trials reported comparable baseline and post‐intervention data for the intervention and control groups and were included in the meta‐analysis. In nine studies, the intervention group performed a cardiovascular training programme. Five papers described a combined training programme. Two trials executed whole‐body vibration training programmes, and one publication proposed balance training as the primary intervention. According to the meta‐analysis results, the reviewed studies proposed exercise modalities that, in comparison with the activities performed by the participants' in the respective control groups, did not have a greater impact on the variables assessed.

**Conclusions:**

While physical exercise can contribute to adiposity‐related anthropometric parameters in people with mild and moderate ID, these findings show that exercise alone is not sufficient to manage obesity in this population. Multicomponent interventions appear to be the best choice when they incorporate dietary deficit, physical activity increase and behaviour change strategies. Finding the most effective modality of physical exercise can only aid weight loss interventions. Future research would benefit from comparing the effects of different exercise modalities within the framework of a multicomponent weight management intervention.

## Introduction

Overweight and obesity, defined as a body mass index (BMI) over 25 or 30 kg/m^2^, respectively, are an excessive fat accumulation (i.e. adiposity) that is associated with poor health outcomes, including an increased risk of incidence and mortality from cancers (Parra‐Soto *et al*. 2021) and cardiovascular diseases (Dwivedi *et al*. 2020). Because of the important health burden and the economic costs to the healthcare systems worldwide derived from obesity, it is a health condition that needs treatment and, more importantly, prevention (Meldrum *et al*. 2017).

Excessive adiposity is largely due to a chronic imbalance between energy intake and energy expenditure favouring the former. Strategies directed at reducing its prevalence should have as a priority to create a negative energy balance. To this aim, exercise promotion plays a key role (Petridou *et al*. 2019). Indeed, exercise can help modify several adiposity‐related anthropometric parameters linked to obesity (i.e. BMI, fat mass and waist circumference) associated with morbidity and mortality (Huxley *et al*. 2010). Internationally recognised guidelines support increasing physical activity (PA) as an integral part of a lifestyle intervention for weight loss, but that this must be combined with a dietary change that produces a calorie deficit and with behaviour change strategies (Scottish Intercollegiate Guidelines Network 2010; National Institute for Health and Care Excellence 2014a,b).

Consequently, there are currently different systematic reviews and meta‐analyses to provide scientific evidence about the benefits of exercise on obese people and the training modalities that are more suitable for them. These pieces of research are generally focused on children (García‐Hermoso *et al*. 2018), adults (Kim *et al*. 2019) and elderly persons (Martínez‐Amat *et al*. 2018) with obesity or in individuals with co‐morbidities related to this condition (Ostman *et al*. 2017). However, research of this kind around people with intellectual disability (ID) has been sparse. This is a population often neglected, despite being at high risk of developing obesity and associated chronic health conditions (Hsieh *et al*. 2014).

Compared with the general population, the prevalence of obesity is much higher in persons with ID, in both adults (Doody & Doody 2012) and youth (Hinckson *et al*. 2013). Obesity leads to a significant contribution to the reduced life expectancy and increased health needs in these individuals (Melville *et al*. 2007). Therefore, there is a need for actions to reduce its prevalence. Obesity has multifactorial causes and decreased resting energy expenditure, and low PA levels have been shown to be determinant factors in those with ID (Bertapelli *et al*. 2016). Accordingly, physical exercise performance takes a prominent role among the lifestyle modifiable factors that can contribute to reducing obesity in this population (Spanos *et al*. 2013).

Efforts to synthesise existing knowledge concerning the effects of exercise interventions on obesity in people with ID have been made (Conrad & Knowlden 2020). Hamilton et al. (2007) reviewed the scientific evidence on the effectiveness of interventions for obesity in ID and indicated that PA interventions proved to be effective in the short term and should be considered an essential component of a weight loss programme. However, they specifically focused on adults with ID. In addition, most of the studies found had significant methodological problems that prevented them from extracting solid conclusions. Similarly, Maïano et al. (2014) reviewed the effectiveness of lifestyle interventions targeting changes in body weight and found that PA programmes were somewhat effective in provoking changes in body weight and composition. The authors focused on young men with ID, and they also observed serious limitations in the reviewed studies, such as small sample sizes or methodological flaws that led to inconclusive findings. Another systematic review and meta‐analysis of randomised controlled trials (RCTs) by Harris et al. (2015) indicated that PA interventions did not significantly change body weight or BMI on young adults with ID, and its effects on other measures of body composition were inconsistent. The generalisability of their findings was limited because of the heterogeneity of the sample population in age ranges and the fact that no comparison on the effects of different PA programmes could be performed. Indeed, the authors highlighted that published RCTs were inadequate to form firm conclusions. More recently, the systematic review and meta‐analysis by Kapsal et al. (2019) showed that PA generally had a positive impact on physical and psychological outcomes, but not in the case of BMI. However, certain methodological aspects such as excluding adults from their analysis and an insufficient number of RCTs that led to a considerable risk of bias limited the strength of the obtained findings. Finally, Casey & Rasmussen (2013) did review the literature on the effects of exercise on both young and adults with ID. They found that although exercise interventions appear effective at maintaining fat levels, they seemed to be particularly ineffective at reducing body fat percentage. These results were limited because the authors conducted a scoping review; thus, there was a lack of uniformity in study design and measurement, and no quality appraisal was performed. Besides, body fat was the only adiposity‐related outcome analysed.

Another important aspect to highlight is that BMI is an indirect marker of adiposity that does not consider body composition (i.e. fat and lean mass). It has been reported that anthropometric proxies for central obesity, such as waist circumference, are better predictors of health risks (Janssen *et al*. 2004; Schneider *et al*. 2010). Furthermore, it has been suggested a higher risk of mortality for high waist circumference compared with high BMI (Yusuf *et al*. 2005; Pischon *et al*. 2008; Staiano *et al*. 2012). The body fat percentage, a direct measure of body fat, is associated with coronary heart disease (Lavie *et al*. 2009), a leading cause of death in developed countries (World Health Organization 2020), and reflects better the serum lipid profile and related metabolic alterations than the anthropometric surrogates (Nagaya *et al*. 1999). In spite of this, no previous systematic reviews have performed a meta‐analysis comparing the effects of different types of physical exercise on these different adiposity‐related parameters in people with ID.

Altogether, it seems that no systematic review and meta‐analysis of RCTs on the effects of PA when performed as a sole intervention on obesity in both children and adults with ID and that also examines the impact of different exercise modalities has been published so far. Therefore, this review aims to answer the following research questions:

- Is physical exercise an accurate strategy to attenuate obesity through its effects on adiposity‐related anthropometric parameters in people with ID?
- What is the methodological quality of the studies that have addressed this topic?
- Is there any physical exercise modality that should be specifically prescribed for this aim in this population?

## Methods

The systematic review and meta‐analysis was conducted in accordance with the Preferred Reporting Items for Systematic Reviews and Meta‐Analysis (PRISMA) guidelines (Moher *et al*. 2009). The review protocol was registered at the PROSPERO database (registration number CRD42020162080).

### Search strategy

A systematic search of randomised controlled trials (RCT) that examined the effects of different PA interventions on adiposity‐related anthropometric parameters in people with ID until July 2019 was performed. Articles from PubMed, Scopus, SPORTDiscus, CINAHL and the Cochrane Library were identified. The search adhered to the population, intervention, comparison and outcome strategy. Only terms regarding the population and the intervention, in a combination of standardised MeSH and free‐text terms, according to recommendations from the Cochrane Handbook for Systematic Reviews of Interventions (Higgins & Green 2008), were used. Consequently, the search syntax was developed as follows: (exercise OR ‘physical activity’ OR ‘physical exercise’) AND (‘intellectual disability’ OR ‘mental retardation’ OR idiocy).

### Eligibility criteria and study selection

The inclusion criteria were as follows: (1) RCT based on exercise or PA programmes, (2) sample entirely made up of individuals with ID and (3) studies analysing the effects of the intervention on adiposity‐related anthropometric parameters (BMI, waist circumference, waist–hip ratio, fat percentage or body weight). Investigations that carried out interventions combined with other types of non‐exercise therapies or that were merely based on the promotion of PA, as well as research not published in English, French, Portuguese or Spanish in peer‐reviewed journals, were excluded.

Titles and abstracts of search results for key criteria were screened, and then full‐text potentially relevant articles for inclusion were assessed. Also, the reference lists of relevant studies for any additional suitable publications were screened. Two authors (M. A. S.‐L. and J. S.‐B.) independently assessed eligibility, with discrepancies resolved through discussion with a third author (C. A. P.).

### Data extraction

One researcher (J. S.‐B.) extracted information using a predeveloped form comprising participants' characteristics, exercise programmes, outcomes and measurement tools, and main findings from the original works, and two investigators (M. A. S.‐L. and C. A. P.) confirmed the accuracy of data extraction. The data extraction procedure was not blind, as the names of the authors of the selected studies and the title of the journals in which they were published were identifiable. Missing data were obtained by contacting the study authors whenever possible.

### Methodological quality assessment

The information on the quality of RCTs was retrieved directly from PEDro, the Physiotherapy Evidence Database. If a trial was not included in PEDro, two authors (M. A. S.‐L. and J. S.‐B.) appraised its quality using the PEDro scale. A third author (C. A. P.) provided advice to reach a consensus in case of disagreement. The PEDro scale rates internal study validity and the presence of replicable statistical information on a scale from 0 to 10. The suggested cut‐off points to categorise studies by quality were excellent (9–10), good (6–8), fair (4–5) and poor (≤3) (Silverman *et al*. 2012).

### Data analysis

A meta‐analysis to measure post‐intervention changes between exercise intervention groups and control groups unassigned to any exercise programmes was used. The standardised mean differences (SMDs) and their 95% confidence intervals (CIs) were calculated to assess the change in each outcome. The SMD was computed using intervention and control group sample sizes, baseline and post‐intervention mean, and standard deviation (SD) for each selected outcome measure. To obtain the change from baseline and post‐intervention SD, the following formula was used: SD_change_ = √(SD^2^_baseline_ + SD^2^_post‐intervention_) − (2 × Corr × SD^2^_baseline_ × SD^2^_post‐intervention_), where Corr = 0.5. The value for Corr was imputed on the assumption of moderate correlation between baseline and post‐intervention measurements (Gates *et al*. 2013; Northey *et al*. 2018). Multiple publications from the same trial were identified in order to avoid double counting the same sample of participants (Senn 2009).

Both fixed‐effect and random‐effect models were applied to determine the pooled effects, considering the first if *I* ^2^ heterogeneity was <30% or the second if higher (DerSimonian & Laird 1986). Forest plots displaying SMDs and 95% CIs to compare the effects between the intervention and control groups were utilised. The SMDs were considered as significant when their 95% CIs excluded zero, while pooled SMD values were evaluated as small (≤0.2), medium (0.2–0.8) or large effects (≥0.8) (Cohen 1992).

All statistical analyses were performed using stata (version 13; StataCorp LLC, College Station, TX).

## Results

**Figure 1:**
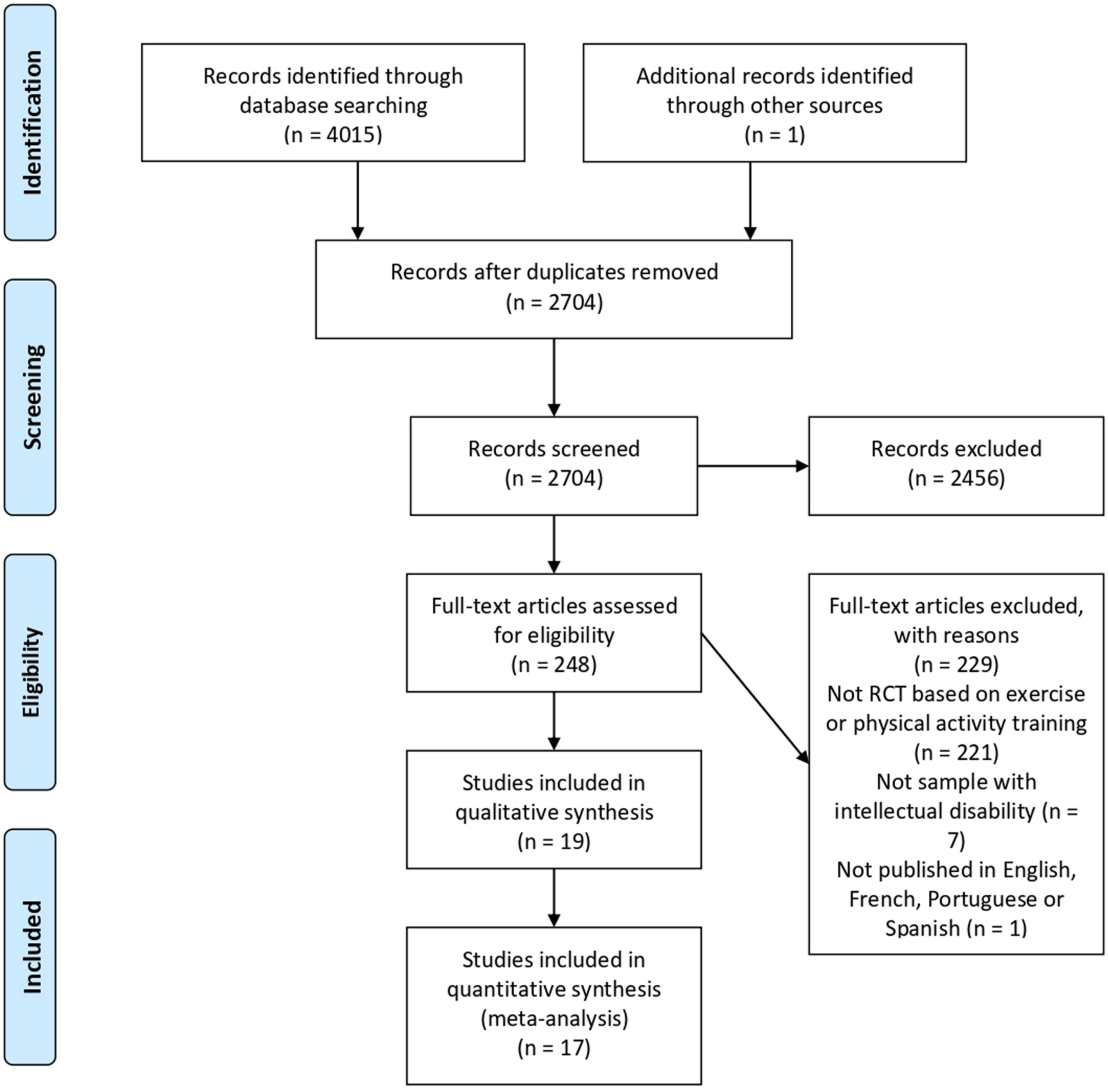
Flow chart of the systematic review process. [Colour figure can be viewed at wileyonlinelibrary.com]

Figure 1 provides a full depiction of the systematic review process. A total of 4015 records from the database search were obtained. After excluding duplicates, 2704 records were identified. Titles and abstracts were screened, and 248 studies for full‐text assessment were retrieved. Finally, 19 RCTs met the full inclusion criteria and were included in the systematic review. Of the total, 17 trials reported comparable baseline and post‐intervention data for both the intervention and control groups and were included in the meta‐analysis. From these, three publications were derived from the same sample (Ordoñez *et al*. 2013; Ordonez *et al*. 2014; Rosety‐Rodriguez *et al*. 2014).

### Design and samples

A total of nine studies included children and/or adolescents (10–19 years), and 10 investigations included adults (18–70 years).

Fourteen publications (Rimmer *et al*. 2004; Calders *et al*. 2011; González‐Agüero *et al*., 2011, 2012, 2013; Lin & Wuang 2012; Ordoñez *et al*. 2013; Boer *et al*. 2014; Ferry *et al*. 2014; Ordonez *et al*. 2014; Rosety‐Rodriguez *et al*. 2014; Matute‐Llorente *et al*. 2015; Melville *et al*. 2015; Shields & Taylor 2015; Boer & Moss 2016; Silva *et al*. 2017) provided information regarding ID aetiology and classified as follows: Down syndrome (DS) (*n* = 449), mental ill health (*n* = 33), autism (*n* = 24), epilepsy (*n* = 22) fragile X syndrome (*n* = 18), problem behaviours (*n* = 18), Prader–Willi syndrome (*n* = 6), fetal alcohol syndrome (*n* = 6) and hydrocephalus (*n* = 3).

A total of eight trials informed about the ID level of the sample, identified through specific scales (Stanford–Binet Scale and Wechsler Scale) as mild (Ordoñez *et al*. 2013; Ordonez *et al*. 2014; Rosety‐Rodriguez *et al*. 2014; Melville *et al*. 2015), moderate (Lin & Wuang 2012; Melville *et al*. 2015; Mikolajczyk & Jankowicz‐Szymanska 2015), mild to moderate (Ozmen *et al*. 2007; Shields & Taylor 2015) and severe (Melville *et al*. 2015). When ID level was not reported, ID condition was determined through medical diagnosis (i.e. DS and Prader–Willi syndrome) (Rimmer *et al*. 2004; Calders *et al*. 2011; González‐Agüero *et al*., 2011, 2012, 2013; Boer *et al*. 2014; Ferry *et al*. 2014; Matute‐Llorente *et al*. 2015; Boer & Moss 2016; Silva *et al*. 2017) and/or by stating participants' intelligent quotient (Calders *et al*. 2011; Boer *et al*. 2014). One paper did not report how the ID was ascertained (Kim 2017).

### Methodological quality

Table 1 shows the methodological quality, which was fair in 13 RCTs (Rimmer *et al*. 2004; González‐Agüero *et al*., 2011, 2012, 2013; Ordoñez *et al*. 2013; Boer *et al*. 2014; Ferry *et al*. 2014; Ordonez *et al*. 2014; Rosety‐Rodriguez *et al*. 2014; Matute‐Llorente *et al*., 2015, 2016; Mikolajczyk & Jankowicz‐Szymanska 2015; Kim 2017), good in five studies (Calders *et al*. 2011; Lin & Wuang 2012; Melville *et al*. 2015; Shields & Taylor 2015; Silva *et al*. 2017) and excellent in one publication (Boer & Moss 2016) (see Table 1 for full quality appraisal criteria). A 100% of trials accomplished item 1 (random allocation) and item 10 (point estimates and variability), while 95% and 89.5% of investigations fulfilled item 9 (between‐group comparisons) and item 3 (baseline comparability), respectively. The items 7 (adequate follow‐up), 6 (blind assessors), 8 (intention‐to‐treat analysis) and 2 (concealed allocation) had a percentage of achievement of 53%, 37%, 26% and 21%, respectively. Only one paper presented a positive result in item 4 (blind participants) (5%), and no RCT attained item 5 (blind therapists).

**Table 1:** PEDro results of the methodological quality evaluation of the included studies

Study	PEDro items										Score	Quality
	1	2	3	4	5	6	7	8	9	10		
Boer & Moss (2016)	+	+	+	+	−	+	+	+	+	+	9/10	Excellent
Lin & Wuang (2012)	+	−	+	−	−	+	+	+	+	+	7/10	Good
Melville *et al*. (2015)	+	+	+	−	−	+	−	+	+	+	7/10	Good
Shields & Taylor (2015)	+	+	−	−	−	+	+	+	+	+	7/10	Good
Silva *et al*. (2017)	+	+	+	−	−	+	+	−	+	+	7/10	Good
Calders *et al*. (2011)	+	−	+	−	−	+	+	−	+	+	6/10	Good
Ordonez *et al*. (2014)	+	−	+	−	−	+	−	−	+	+	5/10	Fair
Rimmer *et al*. (2004)	+	−	+	−	−	−	+	−	+	+	5/10	Fair
Boer *et al*. (2014)	+	−	+	−	−	−	+	−	+	+	5/10	Fair
González‐Agüero *et al*. (2011)	+	−	+	−	−	−	+	−	+	+	5/10	Fair
González‐Agüero *et al*. (2012)	+	−	+	−	−	−	+	−	+	+	5/10	Fair
Rosety‐Rodriguez *et al*. (2014)	+	−	+	−	−	−	+	−	−	+	4/10	Fair
Ordoñez *et al*. (2013)	+	−	+	−	−	−	−	−	+	+	4/10	Fair
Matute‐Llorente *et al*. (2015)	+	−	+	−	−	−	−	−	+	+	4/10	Fair
Ferry *et al*. (2014)	+	−	+	−	−	−	−	−	+	+	4/10	Fair
González‐Agüero *et al*. (2013)	+	−	+	−	−	−	−	−	+	+	4/10	Fair
Mikolajczyk & Jankowicz‐Szymanska (2015)	+	−	+	−	−	−	−	−	+	+	4/10	Fair
Ozmen *et al*. (2007)	+	−	−	−	−	−	+	−	+	+	4/10	Fair
Kim (2017)	+	−	+	−	−	−	−	−	+	+	4/10	Fair

1: random allocation; 2: concealed allocation; 3: baseline comparability; 4: blind participants; 5: blind therapists; 6: blind assessors; 7: adequate follow‐up; 8: intention‐to‐treat analysis; 9: between‐group comparisons; and 10: point estimates and variability.

### Intervention characteristics

Table 2 displays the characteristics of the interventions.

**Table 2:** Summary of characteristics and results of included studies

Study	Sample characteristics	Intervention	Outcomes (measurement tool)	Results
Ordonez *et al*. (2014)	Sample size (*n* pre/post; sex): 20/20; 20 women Distribution; age (years) (mean ± SD): IG: *n* = 11; 24.7 ± 3.6 CG: *n* = 9; 25.1 ± 3.9 BMI (kg/m^2^) (mean ± SD; range): IG: 30.2 ± 0.9; 30.0–31.4 CG: 30.7 ± 0.8; 30.1–31.9 IQ, *score* (mean; range): NR; 50–69 ID level (*n*; scale): mild (all; Stanford–Binet Scale) ID cause (*n*): Down syndrome (all)	Length: 10 weeks IG: Activity: aerobic training programme Volume: 10–15 min of warm‐up, 30–40 min of treadmill exercise (increasing 2 min and 30 s every 2 weeks) and 5–10 min of cooling down Frequency: 3 times per week Intensity: 55–65% of HR_peak_ CG: Non‐exercise training programme	BMI (kg/m^2^) Waist circumference (anthropometric tape) Waist‐to‐hip ratio Fat mass (bioelectrical impedance analysis)	IG adherence (%): NR Significant results: the IG reduced the waist circumference (94.7 ± 3.3 cm pre vs. 91.5 ± 3.1 cm post), waist‐to‐hip ratio (1.12 ± 0.001 pre vs. 1.00 ± 0.001 post) and percentage of fat mass (38.9 ± 4.0% pre vs. 35.0 ± 3.8% post) in the intra‐group analyses
Rosety‐Rodriguez *et al*. (2014)	Sample size (*n* pre/post; sex): 20/20; 20 women Distribution; age (years) (mean ± SD): IG: *n* = 11; 24.7 ± 3.6 CG: *n* = 9; 25.1 ± 3.9 BMI (kg/m^2^) (mean ± SD): IG: 30.2 ± 0.9 CG: 30.7 ± 0.8 IQ, *score* (mean; range): NR; 50–69 ID level (*n*; scale): mild (all; Stanford–Binet Scale) ID cause (*n*): Down syndrome (all)	Length: 10 weeks IG: Activity: aerobic training programme Volume: 10–15 min of warm‐up, main part of 30–40 min (increasing 2 min and half each 2 weeks) and 5–10 min of cooling‐down period Frequency: 3 sessions per week Intensity: 55–65% of HR_peak_ (increasing 2.5% every 2 weeks) CG: Non‐exercise training programme	Waist circumference (anthropometric tape) Fat mass (bioelectrical impedance analysis)	IG adherence (%): NR Significant results: the IG reduced waist circumference (94.7 ± 3.3 cm pre vs. 91.5 ± 3.1 cm post) and percentage of fat mass (38.9 ± 4.0% pre vs. 35.0 ± 3.8% post) in the intra‐group analyses
Ordoñez *et al*. (2013)	Sample size (*n* pre/post; sex): 20/20; 20 women Distribution; age (years) (mean ± SD): IG: *n* = 11; 24.7 ± 3.6 CG: *n* = 9; 25.1 ± 3.9 BMI: NR IQ, *score* (mean; range): NR; 50–69 ID level (*n*; scale): mild (all; Stanford–Binet Scale) ID cause (*n*): Down syndrome (all)	Length: 10 weeks IG: Activity: aerobic training exercises Volume: 10–15 min of warm‐up, 30–40 min, increasing by 2.5 min every 2 weeks and 5–10 min of cooling‐down period Frequency: 3 times per week Intensity: 55–65% of HR_peak_ (increasing 2.5% every 2 weeks) CG: Non‐exercise training programme	Waist circumference (anthropometric tape) Waist‐to‐hip ratio Fat mass (bioelectrical impedance analysis)	IG adherence (%): NR Significant results: the IG reduced waist circumference (94.7 ± 3.3 cm pre vs. 91.5 ± 3.1 cm post), waist‐to‐hip ratio (1.12 ± 0.006 pre vs. 1.00 ± 0.005 post) and percentage of fat mass (38.9 ± 4.6% pre vs. 35.0 ± 4.2% post) in the intra‐group analyses
Lin & Wuang (2012)	Sample size (*n* pre/post; sex): 92/92; 49 women Distribution; age (years) (mean ± SD): IG: *n* = 46; 15.6 ± 3.6 CG: *n* = 46; 14.9 ± 3.9 BMI (kg/m^2^) (mean ± SD): IG: 29.5 ± 8.8 CG: 30.2 ± 7.6 IQ, *score* (mean): 52 (IG), 53 (CG) ID level (*n*; scale): moderate (NR; Wechsler Intelligence Scale for Children) ID cause (*n*): Down syndrome (all)	Length: 6 weeks IG: Activity: treadmill and virtual reality‐based exercise Volume: 5 min of treadmill exercise and 20 min of virtual reality‐based exercise, 10 min of break in between Frequency: 3 sessions per week Intensity: NR CG: Non‐exercise training group	Weight (specific questionnaire) BMI (kg/m^2^)	IG adherence (%) (mean): 100 Significant results: the IG decreased weight (52.2 ± 7.2 kg pre vs. 49.8 ± 6.6 kg post) in comparison with the CG after the intervention
Matute‐Llorente *et al*. (2015)	Sample size (*n* pre/post; sex): 30/25; 8 women Distribution; age (years) (mean ± SD): IG: *n* = 11; 15.2 ± 2.5 CG: *n* = 14; 15.5 ± 3.0 BMI (kg/m^2^) (mean ± SD): IG: 21.4 ± 2.8 CG: 23.5 ± 5.0 IQ, *score*: NR ID level: NR ID cause (*n*): Down syndrome (all)	Length: 20 weeks IG: Activity: whole‐body vibration training Volume: 15–20 min of total training time, using 10 repetitions of 30–60 s of vibration training with 60 s of rest between repetitions Frequency: 3 sessions per week Intensity: 25 to 30 Hz CG: Non‐exercise programme; daily life activities	Weight (stadiometer) BMI (kg/m^2^)	IG adherence (%): NR Significant results: NR
Melville *et al*. (2015)	Sample size (*n* pre/post; sex): 102/82; 45 women Distribution; age (years) (mean ± SD): IG: *n* = 54; 44.9 ± 13.5 CG: *n* = 48; 47.7 ± 12.3 BMI (kg/m^2^) (mean ± SD): IG: 32.3 ± 7.3 CG: 32.6 ± 7.4 IQ, *score*: NR ID level (*n*; scale): mild, moderate and severe (NR; NR) ID cause (*n*): mental ill health (33), problem behaviours (18), epilepsy (10)	Length: 12 weeks IG: Activity: walking Volume: increase their daily walking time by 30 min (around 3000 steps) Frequency: 5 days/week Intensity: NR CG: Invited to take part in IG programme at the end of the 12‐week waiting list period	BMI (kg/m^2^) Waist circumference (NR)	IG adherence (%): NR Significant results: NR
Rimmer *et al*. (2004)	Sample size (*n* pre/post; sex): 52/52; 29 women Distribution; age (years) (mean ± SD): IG: *n* = 30; 38.6 ± 6.2 CG: *n* = 22; 40.6 ± 6.5 BMI (kg/m^2^) (mean ± SD): IG: 35.2 ± 8.7 CG: 33.9 ± 7.6 IQ, *score*: NR ID level: NR ID cause (*n*): Down syndrome (all)	Length: 12 weeks IG: Activity: strength and endurance training Volume: 30–45 min of cardiovascular training and 15–20 min of muscular strength and endurance training; 3–5 min of warm‐up and 3–5 min of cool‐down Frequency: 3 sessions per week Intensity: for endurance training, HR zone previously prescribed and 50–70% V̇O₂_peak_ (week 5). For strength training, 70% 1RM and the weight was increased by 10% of their 1RM CG: Non‐exercise training programme	Weight (NR) BMI (NR)	IG adherence (%): NR Significant results: the IG reduced weight after the intervention (80.5 ± 20 kg pre vs. 79.5 ± 19.9 kg post)
Shields & Taylor (2015)	Sample size (*n* pre/post; sex): 16/16; 8 women Distribution; age (years) (mean ± SD): IG: *n* = 8; 21.6 ± 3.4 CG: *n* = 8; 21.2 ± 3.2 BMI (kg/m^2^) (mean ± SD): IG: 32.2 ± 6.3 CG: 25.1 ± 3.0 IQ, *score*: NR ID level (*n*; scale): mild to moderate (all; parent report) ID cause (*n*): Down syndrome (all)	Length: 8 weeks IG: Activity: walking Volume: 150 min/week. Two 45‐min walking sessions each week. The other 60 min was individually tailored to the circumstances of the participant Frequency: 2 sessions per week Intensity: moderate CG: Social activities	Weight (weighting scale) Waist circumference (tape measure)	IG adherence (%) (mean): 96 Significant results: NR
Silva *et al*. (2017)	Sample size (*n* pre/post; sex): 27/25; NR women Distribution; age (years) (range): IG: *n* = 14; 18–60 CG: *n* = 13; 18–60 BMI (kg/m^2^) (mean ± SD): IG: 32.2 ± 6.4 CG: 32.0 ± 6.9 IQ, *score*: NR ID level: NR ID cause (*n*): Down syndrome (all)	Length: 8 weeks IG: Activity: balance, isometric strength exercises and aerobic endurance using a Wii console Volume: 60 min Frequency: 3 sessions per week Intensity: NR CG: Usual daily activities (treatment‐as‐usual) on their occupational centre such as vocational rehabilitation, life‐skill training and art‐related activities	Weight (segmental body composition analyser) BMI (segmental body composition analyser) Waist circumference (steel anthropometric tape) Fat mass (segmental body composition analyser)	IG adherence (%): NR Significant results: NR
Boer *et al*. (2014)	Sample size (*n* pre/post; sex): 54/46; 16 women Distribution; age (years) (mean ± SD): IG1: *n* = 17; 18 ± 3.2 IG2: *n* = 15; 16.7 ± 3.6 CG: *n* = 14; 17.4 ± 2.4 BMI (kg/m^2^) (mean ± SD): IG1: 28.4 ± 4.7 IG2: 27.5 ± 2.7 CG: 26.9 ± 3.2 IQ, *score* (mean): 59.2 (IG1), 57.3 (IG2), 59.1 (CG) ID level: NR ID cause (*n*): fragile X syndrome (NR), fetal alcohol syndrome (NR), Prader–Willi syndrome (NR), hydrocephalus (NR), pervasive developmental disorder (NR), Sotos syndrome and Steinert syndrome (NR), severe autism (NR), epilepsy or attention deficit hyperactivity disorder (NR)	Length: 15 weeks IG1: Activity: continuous aerobic and sprint interval training Volume: 40 min. Warm‐up of 5 min, 10 min of sprint interval block, 10 min of continuous aerobic exercise, 10 min of sprint interval block and 5 min of cooling down Frequency: 2 times per week Intensity: each sprint interval block consisted of 10 sprint bouts (>100 r.p.m.) of 15 s at a resistance matching with the ventilatory threshold, alternated with 45‐s relative rest (50 r.p.m. at ventilatory threshold) (first 7 weeks). The intensity of sprinting and relative rest was increased up to 110% of ventilatory threshold (weeks 8 to 15) IG2: Activity: aerobic training Volume: 5 min of warm‐up, 10 min of cycling, 10 min of walking/running, 10 min of stepping and 5 min of cooling down Frequency: NR Intensity: 10 min at an HR similar to the HR at ventilatory threshold (60 r.p.m.), which was increased to 110% of ventilatory threshold from week 8 onwards CG: Non‐exercise training programme. Usual everyday scholar activities without supervised exercise training	Weight (digital balance scale) BMI (kg/m^2^) Waist circumference (tape metre) Fat mass (bioelectrical impedance analysis)	IG adherence (%): NR Significant results: after the intervention, the IG2 showed a reduction in waist circumference (95.9 ± 9.6 cm pre vs. 93.4 ± 9.6 cm post) and fat mass (32.3 ± 7.0% pre vs. 31.3 ± 6.6% post) compared with the CG. Fat mass was more decreased in the IG1 (34.2 ± 6.9% pre vs. 30.4 ± 7.0% post) compared with the IG2
Boer & Moss (2016)	Sample size (*n* pre/post; sex): 46/42; 17 women Distribution; age (years) (mean ± SD): IG1: *n* = 13; 30.0 ± 7.4 IG2: *n* = 13; 34.2 ± 9.2 CG: *n* = 16; 36.6 ± 8.4 BMI (kg/m^2^) (mean ± SD): IG1: 29.3 ± 4.0 IG2: 30.6 ± 6.1 CG: 31.2 ± 4.6 IQ, *score*: NR ID level: NR ID cause (*n*): Down syndrome (all)	Length: 12 weeks IG1: Activity: sprint training (19–30 s all out) and low‐intensity walking or cycling (90 s) Volume: 5 min of warm‐up, 20 min of training and 5 min of cool‐down Frequency: 3 times per week Intensity: 1:3 work–rest ratio IG2: Activity: continuous cycling or walking Volume: 5 min of warm‐up, 20 min of training and 5 min of cool‐down Frequency: 3 times per week Intensity: 70–80% to 85% VO₂_peak_ CG: Non‐exercise programme	Weight (calibrated electronic scale) BMI (kg/m^2^) Waist circumference (flexible steel tape) Fat mass (bioelectrical impedance analysis)	IG adherence (%) (mean): 95 (IG1), 96 (IG2) Significant results: in the IG1, weight (71.7 ± 8.4 kg pre vs. 69.4 ± 8.3 kg post) and BMI (29.0 ± 4.0 kg/m^2^ pre vs. 28.5 ± 4.0 kg/m^2^ post) were more decreased compared with the CG after intervention. These variables also decreased more in the IG2 (weight, 70.2 ± 14.6 kg pre vs. 69.2 ± 14.6 kg post; BMI, 30.6 ± 6.1 kg/m^2^ pre vs. 30.2 ± 6.3 kg/m^2^ post) compared with the CG. Weight decreased more in the IG2 compared with the CG
Calders *et al*. (2011)	Sample size (*n* pre/post; sex): 45/45; 27 women Distribution; age (years) (mean ± SD): IG1: *n* = 15; 42 ± 7.5 IG2: *n* = 15; 42 ± 9.3 CG: *n* = 15;43 ± 11.4 BMI (kg/m^2^) (mean ± SD): IG1: 24.0 ± 3.9 IG2: 25.7 ± 4.1 CG: 22.3 ± 3.4 IQ, *score* (mean; range): 56 (IG1), 58 (IG2), 53 (CG); 45–70 ID level: NR ID cause (*n*): severe autism (24), fragile X syndrome (18), mild epilepsy (12), fetal alcohol syndrome (6), Prader–Willi syndrome (6), hydrocephalus (3)	Length: 20 weeks IG1: Activity: strength and endurance exercises Volume: 70 min; 5 min of warm‐up, 10 min of cycling, 10 min of strength training (biceps brachii and triceps brachii), 10 min of stepping, 10 min of strength training (quadriceps and hamstrings), 10 min of running, 10 min of functional training of abdominal and back muscles and 5 min of cooling down Frequency: 2 times per week Intensity: 90% of ventilatory anaerobic threshold to 100% after 10 sessions and 110% after 20 sessions (endurance training). 1RM (strength training) IG2: Activity: endurance exercises Volume: 70 min. Protocol was similar to the IG1, but the strength components were replaced by cycling, stepping and walking/running Frequency: 2 times per week Intensity: 90% of ventilatory anaerobic threshold to 100% after 10 sessions and 110% after 20 sessions CG: Non‐exercise programme. Daily activities without supervised exercise training	Weight (digital balance scale) BMI (kg/m^2^) Waist circumference (tape measure) Fat mass (formula of Kyle) Lean mass (formula of Kyle)	IG adherence (%) (total): 90 Significant results: NR
Ferry *et al*. (2014)	Sample size (*n* pre/post; sex): 42/42; 18 women Distribution; age (years) (mean ± SD): IG: *n* = 20; 16.0 ± 1.8 CG: *n* = 22; 16.9 ± 1.5 BMI (kg/m^2^) (mean ± SD): IG: 25.2 ± 6.7 CG: 27.1 ± 6.7 IQ, *score*: NR ID level: NR ID cause (*n*): Down syndrome (all)	Length: 12 months IG: Activity: extracurricular physical activities focused on the development of general physical qualities Volume: 60 min. A total of 15 min of a warm‐up, main part of 40 min and 5 min of recovery Frequency: 2 times per week Intensity: moderate to vigorous CG: Non‐exercise training group	Weight (balance‐beam scale) BMI (kg/m^2^) Fat mass (Siri equation)	IG adherence (%): NR Significant results: the IG decreased the fat mass percentage (33.5 ± 5.% pre vs. 33.1 ± 5.2% post) after the intervention in the intra‐group analysis
González‐Agüero *et al*. (2013)	Sample size (*n* pre/post; sex): 30/24; 11 women Distribution; age (years) (mean ± SD): IG: *n* = 16; 15.3 ± 2.6 CG: *n* = 14; 15.8 ± 3.0 BMI (kg/m^2^) (mean ± SD): IG: 21.5 ± 2.8 CG: 24.1 ± 4.7 IQ, *score*: NR ID level: NR ID cause (*n*): Down syndrome (all)	Length: 20 weeks IG: Activity: whole‐body vibration training Volume: 15–20 min of total training time, using 10 repetitions of 30–60 s of vibration training with 60 s of rest between repetitions Frequency: 3 sessions per week Intensity: 25 to 30 Hz CG: Non‐exercise programme	Weight (stadiometer) BMI (kg/m^2^) Fat mass (dual‐energy X‐ray absorptiometry) Lean mass (dual‐energy X‐ray absorptiometry)	IG adherence (%) (mean ± SD): 69.9 ± 18.7 Significant results: the IG showed a higher per cent declination in fat mass at the upper limbs (0.78 ± 0.06 kg pre vs. 0.75 ± 0.09 kg post) than the control group
González‐Agüero *et al*. (2011)	Sample size (*n* pre/post; sex): 26/25; 13 women Distribution; age (years) (mean ± SD): IG: *n* = 12; 13.7 ± 2.6 CG: *n* = 13; 15.4 ± 2.5 BMI (kg/m^2^) (mean ± SD): IG: 19.6 ± 2.7 CG: 22.4 ± 3.4 IQ, *score*: NR ID level: NR ID cause (*n*): Down syndrome (all)	Length: 21 weeks IG: Activity: combined conditioning and plyometric jump Volume: 5 min warm‐up activities, 10–15 min for the main part of the session and 5 min of cool‐down Frequency: twice a week Intensity: NR CG: Non‐exercise programme	Weight (stadiometer) BMI (kg/m^2^) Fat mass (dual‐energy X‐ray absorptiometry) Lean mass (dual‐energy X‐ray absorptiometry)	IG adherence (%) (mean ± SD): 81.8 ± 9.2 Significant results: BMI decreased in the CG (22.4 ± 3.4 kg/m^2^ pre vs. 22.3 ± 3.2 kg/m^2^ post). Fat mass was also decreased in the CG in the analysis adjusted for height and Tanner stage (whole‐body fat mass, 12.4 ± 1.3 kg pre vs. 12.0 ± 1.3 kg post)
González‐Agüero *et al*. (2012)	Sample size (*n* pre/post; sex): 28/27; 13 women Distribution; age (years) (mean ± SD): IG: *n* = 14; 13.8 ± 2.6 CG: *n* = 13; 15.5 ± 2.6 BMI (kg/m^2^) (mean ± SD): IG: 19.6 ± 2.7 CG: 22.4 ± 3.4 IQ, *score*: NR ID level: NR ID cause (*n*): Down syndrome (all)	Length: 21 weeks IG: Activity: combined conditioning and plyometric jump Volume: 5 min of warm‐up activities, 10–15 min for the main part of the session and 5 min of cool‐down Frequency: twice a week Intensity: NR CG: Non‐exercise programme	Weight (stadiometer) BMI (kg/m^2^)	IG adherence (%) (mean): 83.3 Significant results: NR
Mikolajczyk & Jankowicz‐Szymanska (2015)	Sample size (*n* pre/post; sex): 34/34; 6 women Distribution; age (years) (mean ± SD): IG: *n* = 17; NR CG: *n* = 17; NR Total: 15.06 ± 0.91 BMI (kg/m^2^) (mean ± SD): IG: 24.8 ± 6.2 CG: 24.4 ± 3.3 IQ, *score* (mean ± SD): 45.5 ± 3.39 ID level (*n*; scale): moderate (NR; NR) ID cause: NR	Length: 12 weeks IG: Activity: programme targeted at improving postural balance Volume: 45 min Frequency: 3 sessions per week Intensity: NR CG: Non‐exercise programme	Weight (stadiometer) BMI (kg/m^2^)	IG adherence (%): NR Significant results: the IG decreased weight (65.8 ± 14.0 kg pre vs. 65.0 ± 13.6 kg post) and BMI (24.8 ± 6.2 kg/m^2^ pre vs. 24.3 ± 5.9 kg/m^2^ post) in the intra‐group analyses
Ozmen *et al*. (2007)	Sample size (*n* pre/post; sex): 30/30; NR women Distribution; age (years) (mean ± SD): IG: *n* = 16; 10.9 ± 2.0 CG: *n* = 14; 11.4 ± 2.0 BMI (kg/m^2^) (mean ± SD): IG: 19.5 ± 4.7 CG: 18.0 ± 2.4 IQ, *score*: NR ID level (*n*; scale): mild to moderate (all; NR) ID cause: NR	Length: 10 weeks IG: Activity: strength and endurance exercises Volume: 10 min of warm‐up, 20 min of interval training and 25 min of recreational activities (volleyball, soccer, rope jumping and dodgeball), followed by 5 min of cool‐down period Frequency: 3 times per week Intensity: NR CG: Non‐exercise training programme. They would have normally engaged in their habitual physical activity, which included attending a general physical education class for approximately 2 h/week	Fat mass (manual skinfold callipers)	IG adherence (%): NR Significant results: not found
Kim (2017)	Sample size (*n* pre/post; sex): 24/24; NR women Distribution; age (years) (mean ± SD): IG1: *n* = 8; 19.3 ± 1.2 CG: *n* = 8; 20.2 ± 1.1 BMI: NR IQ, *score*: NR ID level: NR ID cause: NR	Length: 12 weeks IG: Activity: aerobic exercise Volume: two 15‐min exercise periods were performed within 50‐min period Frequency: 5 times per week Intensity: 50–70% HR_max_ CG: Non‐exercise programme or half‐bath treatment	Weight (bioelectrical impedance instrument) Fat mass (bioelectrical impedance instrument)	IG adherence (%): NR Significant results: the IG decreased weight (65.6 ± 1.5 kg pre vs. 61.3 ± 1.6 kg post) and fat mass percentage (32.3 ± 1.6% pre vs. 27.5 ± 1.1% post) compared with the CG after intervention. The CG increased both weight and fat mass percentage

1RM, one‐repetition maximum; BMI, body mass index; CG, control group; HR, heart rate; HR_max_, maximum heart rate; HR_peak_, peak heart rate; ID, intellectual disability; IG, intervention group; IQ, intelligence quotient; NR, not reported; SD, standard deviation; V̇O₂_peak_, peak oxygen uptake.

In nine studies, the intervention group (IG) performed a cardiovascular training programme, and these investigations were classified as ‘cardiovascular exercise’ in the meta‐analysis (Ozmen *et al*. 2007; Lin & Wuang 2012; Boer *et al*. 2014; Ordonez *et al*. 2014; Rosety‐Rodriguez *et al*. 2014; Melville *et al*. 2015; Shields & Taylor 2015; Boer & Moss 2016; Kim 2017). Two publications compared two types of exercise modalities (sprint interval training vs. continuous aerobic training); hence, two IGs were included in these trials (Boer *et al*. 2014; Boer & Moss 2016).

Five papers described a combined training programme. Rimmer et al. (2004) combined strength training and endurance training. González‐Agüero et al. (2012) carried out a plyometric training programme and general conditioning using press‐ups on walls, fitness bands and medicine balls. In contrast, Ferry et al. (2014) employed plyometric jumps, bodybuilding exercises, agility and speed exercises, and gymnastic routines, all in the form of dynamic games. Silva et al. (2017) implemented a combined balance and isometric strength training programme. Finally, one RCT (Calders *et al*. 2011), with two different IGs, conducted strength and endurance training (IG1) and endurance training (IG2).

Two studies executed whole‐body vibration training programmes (González‐Agüero *et al*. 2013; Matute‐Llorente *et al*. 2015), and one investigation proposed balance training as the primary intervention (Mikolajczyk & Jankowicz‐Szymanska 2015). These three trials were included in the meta‐analysis as ‘other interventions’.

The duration of the training programmes ranged between 6 (Lin & Wuang 2012) and 52 weeks (1 year) (Ferry *et al*. 2014) in length, with sessions between 25 (González‐Agüero *et al*. 2011; Lin & Wuang 2012) and 60 min long (Rimmer *et al*. 2004; Ozmen *et al*. 2007; Ordoñez *et al*. 2013; Ferry *et al*. 2014; Ordonez *et al*. 2014; Rosety‐Rodriguez *et al*. 2014), and a frequency ranging from 2 (Calders *et al*. 2011; González‐Agüero *et al*. 2011; Boer *et al*. 2014; Ferry *et al*. 2014) to 3 days/week (Ozmen *et al*. 2007; Lin & Wuang 2012; González‐Agüero *et al*. 2013; Ordoñez *et al*. 2013; Ordonez *et al*. 2014; Rosety‐Rodriguez *et al*. 2014; Matute‐Llorente *et al*., 2015, 2016; Mikolajczyk & Jankowicz‐Szymanska 2015; Boer & Moss 2016; Silva *et al*. 2017).

Regarding exercise intensity prescription, four publications used the participant's maximum heart rate (HR_max_), ranging from 50% to 70% HR_max_ (Ordoñez *et al*. 2013; Ordonez *et al*. 2014; Rosety‐Rodriguez *et al*. 2014; Kim 2017); two papers prescribed exercise intensity based on maximum oxygen consumption (V̇O₂_max_), ranging from 50% to 80% V̇O₂_max_ (Rimmer *et al*. 2004; Boer & Moss 2016); and one RCT opted for the ventilatory anaerobic threshold increasing from 90% up to 110% (Calders *et al*. 2011). The one‐repetition maximum was used to prescribe the intensity of the strength training programme in two studies (Rimmer *et al*. 2004; Calders *et al*. 2011).

None of the investigations with control groups assigned these to any exercise programmes during the intervention, requiring them to avoid exercise participation in 12 publications (Rimmer *et al*. 2004; González‐Agüero *et al*., 2011, 2013; Lin & Wuang 2012; Boer *et al*. 2014; Ferry *et al*. 2014; Ordonez *et al*. 2014; Rosety‐Rodriguez *et al*. 2014; Melville *et al*. 2015; Mikolajczyk & Jankowicz‐Szymanska 2015; Boer & Moss 2016; Kim 2017); or to continue with the daily life activities in four trials (Ozmen *et al*. 2007; Calders *et al*. 2011; Matute‐Llorente *et al*. 2015; Silva *et al*. 2017); or to perform social activities in one paper (Shields & Taylor 2015).

Seven RCTs reported adherence, which ranged between 69.9% and 100% (Calders *et al*. 2011; González‐Agüero *et al*., 2011, 2012, 2013; Lin & Wuang 2012; Shields & Taylor 2015; Boer & Moss 2016).

### Clinical significance of the results

A total of 14 studies reported body weight measurements. Of those, only three studies (Lin & Wuang 2012; Boer & Moss 2016; Kim 2017) reported an average weight loss superior to −3% (ranging between 3.2% and 6.6%), which is considered of clinical relevance (National Institute for Health and Care Excellence 2014b).

### Results of the meta‐analysis

#### Weight

**Figure 2:**
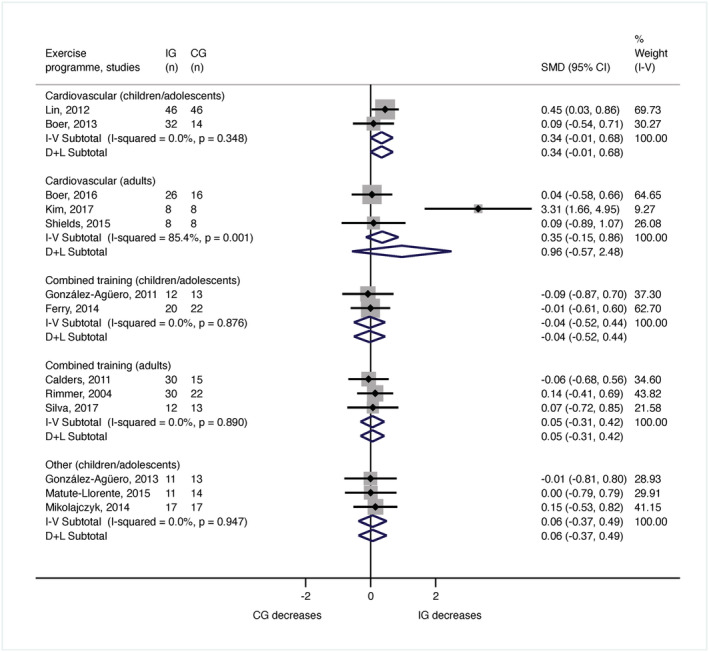
Meta‐analysis for the body weight by age and type of exercise programme. CG, control group; CI, confidence interval; IG, intervention group; SMD, standardised mean difference. [Colour figure can be viewed at wileyonlinelibrary.com]

In the meta‐analysis for body weight, a total of 484 individuals were included (288 children or adolescents and 196 adults). In children and adolescents, seven studies analysed weight (González‐Agüero *et al*., 2011, 2013; Lin & Wuang 2012; Boer *et al*. 2014; Ferry *et al*. 2014; Matute‐Llorente *et al*. 2015; Mikolajczyk & Jankowicz‐Szymanska 2015). The meta‐analysis demonstrated no significant results for cardiovascular training (*n* = 138; SMD = 0.34, 95% CI [−0.01, 0.68]; *I* ^2^ = 0%), combined training (*n* = 67; SMD = −0.04, 95% CI [−0.52, 0.44]; *I* ^2^ = 0%) or other type of exercise (*n* = 83; SMD = 0.06, 95% CI [−0.37, 0.49]; *I* ^2^ = 0%) (Fig. 2). In adults, six investigations assessed weight (Rimmer *et al*. 2004; Calders *et al*. 2011; Shields & Taylor 2015; Boer & Moss 2016; Kim 2017; Silva *et al*. 2017). The meta‐analysis showed no significant results for cardiovascular training (*n* = 74; SMD = 0.35, 95% CI [−0.15, 0.86]; *I* ^2^ = 85.4%) or combined training programmes (*n* = 122; SMD = 0.05, 95% CI [−0.31, 0.42]; *I* ^2^ = 0%) (Fig. 2).

#### Body mass index

**Figure 3:**
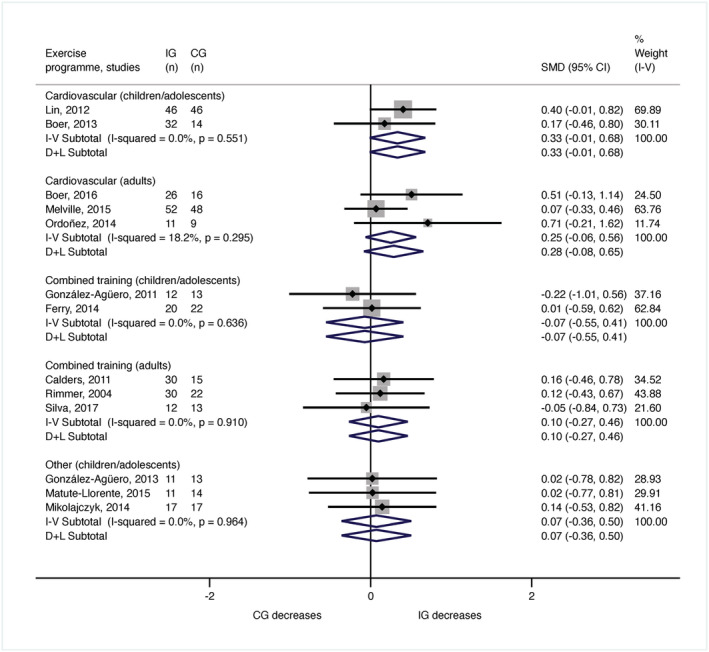
Meta‐analysis for the body mass index by age and type of exercise programme. CG, control group; CI, confidence interval; IG, intervention group; SMD, standardised mean difference. [Colour figure can be viewed at wileyonlinelibrary.com]

The meta‐analysis for BMI comprised 572 participants (288 children or adolescents and 284 adults). The meta‐analysis of the seven trials evaluating the BMI changes in children and adolescents (González‐Agüero *et al*. 2011, 2013; Lin & Wuang 2012; Boer *et al*. 2014; Ferry *et al*. 2014; Matute‐Llorente *et al*. 2015) revealed no significant modifications after cardiovascular training (*n* = 138; SDM = 0.33, 95% CI [−0.01, 0.68]; *I* ^2^ = 0%), or combined training programmes (*n* = 67; SDM = −0.07, 95% CI [−0.55, 0.41]; *I* ^2^ = 0%) or balance and whole‐body vibration training (*n* = 83; SDM = 0.07, 95% CI [−0.36, 0.50]; *I* ^2^ = 0%) (Fig. 3). In adults, six RCTs measured BMI (Rimmer *et al*. 2004; Calders *et al*. 2011; Ordonez *et al*. 2014; Melville *et al*. 2015; Boer & Moss 2016; Silva *et al*. 2017). The meta‐analysis indicated no significant changes in BMI after cardiovascular training (*n* = 162; SDM = 0.25, 95% CI [−0.06, 0.56]; *I* ^2^ = 18.2%) or combined training programmes (*n* = 122; SDM = 0.10, 95% CI [−0.27, 0.46]; *I* ^2^ = 0%) (Fig. 3).

#### Fat mass

**Figure 4:**
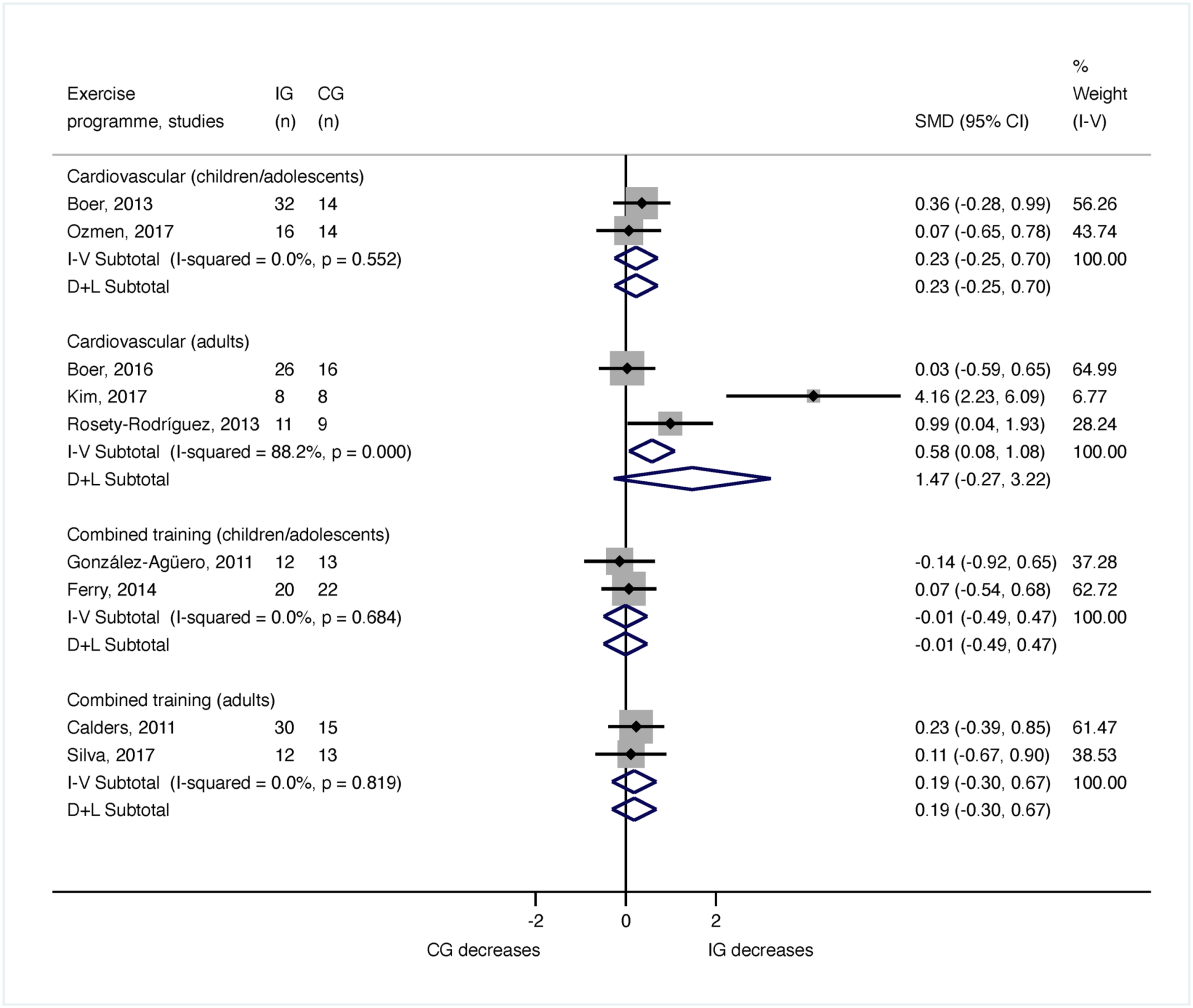
Meta‐analysis for the body fat by age and type of exercise programme. CG, control group; CI, confidence interval; IG, intervention group; SMD, standardised mean difference. [Colour figure can be viewed at wileyonlinelibrary.com]

The meta‐analysis for fat mass incorporated 288 participants (143 children or adolescents and 145 adults). In children and adolescents, fat mass was inspected in four publications (Ozmen *et al*. 2007; González‐Agüero *et al*. 2011; Boer *et al*. 2014; Ferry *et al*. 2014), and the meta‐analysis showed no significant changes after performing cardiovascular training (*n* = 76; SMD = 0.23, 95% CI [−0.48, 0.46]; *I* ^2^ = 0%) or combined training programmes (*n* = 67; SMD = −0.01, 95% CI [−0.49, 0.47]; *I* ^2^ = 0%) (Fig. 4). In adults, body fat was examined in five studies (Calders *et al*. 2011; Rosety‐Rodriguez *et al*. 2014; Boer & Moss 2016; Kim 2017; Silva *et al*. 2017). The results of the meta‐analysis were non‐significant for cardiovascular training (*n* = 78; SMD = 0.58, 95% CI [0.08, 1.08]; *I* ^2^ = 88.2%) or combined training programmes (*n* = 67; SMD = 0.19, 95% CI [−0.30, 0.67]; *I* ^2^ = 0%) (Fig. 4).

#### Waist circumference

**Figure 5:**
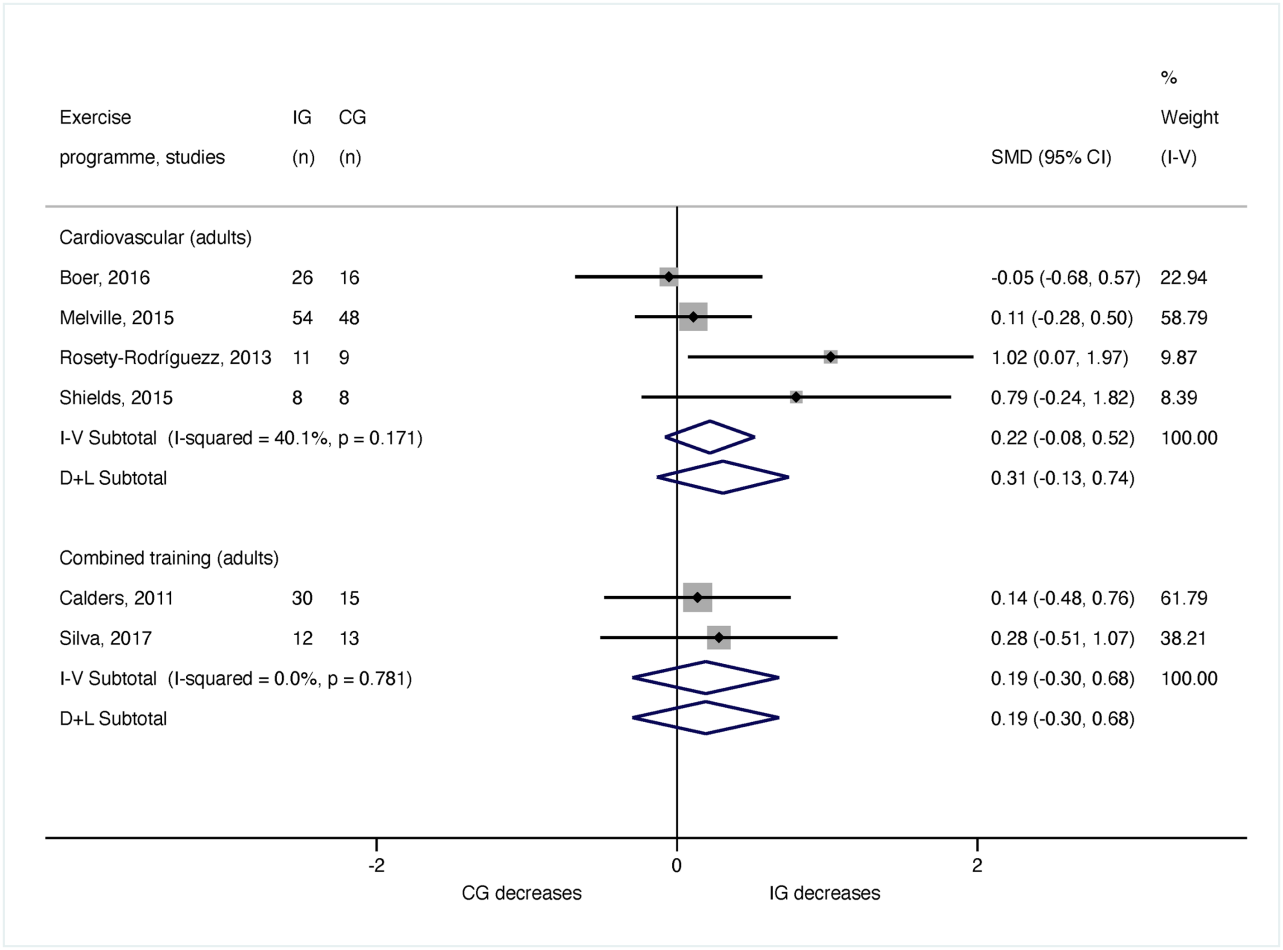
Meta‐analysis for the waist circumference by age and type of exercise programme. CG, control group; CI, confidence interval; IG, intervention group; SMD, standardised mean difference. [Colour figure can be viewed at wileyonlinelibrary.com]

A total of 250 adults from six articles (Calders *et al*. 2011; Rosety‐Rodriguez *et al*. 2014; Melville *et al*. 2015; Shields & Taylor 2015; Boer & Moss 2016; Silva *et al*. 2017) were pooled in the meta‐analysis for waist circumference. There were no significant results for cardiovascular training (*n* = 180; SMD = 0.22, 95% CI [−0.08, 0.52]; *I* ^2^ = 40.1%) or combined training programmes (*n* = 70; SMD = 0.19, 95% CI [−0.30, 0.68]; *I* ^2^ = 0%) (Fig. 5).

#### Sensitivity analysis

The results of the sensitivity analysis, including only aerobic exercise programmes in children or adolescents and adults, exhibited significant improvements for BMI when the analysis was not stratified by age groups (Fig. S1). A trend towards improvement was generally found for all variables (weight, BMI, waist circumference and body fat, Fig. S2).

## Discussion

This systematic review aimed at summarising, critically evaluating and integrating the available scientific evidence regarding the impact of exercise on a number of obesity‐related parameters related in children, adolescents and adults with ID. A considerable number of RCTs ranging from fair to excellent methodological quality were found, and the meta‐analysis included 85% of these trials. Thus, the information provided here can help guide best practices among those therapists and researchers interested in the possibilities and potential impact of prescribing exercise as a weight loss strategy for people with ID.

According to the meta‐analysis results, the reviewed studies proposed exercise modalities that, in comparison with the activities performed by the participants in the respective control groups, did not have a greater impact on the variables assessed. Given that the training programmes were generally well designed, and exercise adherence was usually high, our results would imply that exercise alone does not lead to significant anthropometric and body weight parameter changes. This finding is in line with previous observations indicating that exercise often does not produce significant morphological changes in people with ID (Hamilton *et al*. 2007; Spanos *et al*. 2013; Harris *et al*. 2015; Martínez‐Aldao *et al*. 2019). Moreover, these results reinforce the idea that interventions targeting obesity should focus on reducing energy intake rather than solely on exercise‐induced energy expenditure (Westerterp 2019).

Nevertheless, it is worth mentioning that the sensitivity analysis showed a significant trend revealing that aerobic exercise might contribute to significant changes in obesity‐related parameters. Indeed, when all the investigations that utilised this exercise modality were analysed together (without considering the participants' age), the results were statistically significant. Thus, it is plausible to think that if the reviewed trials had a greater sample size, the meta‐analysis would have detected a significant impact of aerobic exercise on anthropometric and body composition parameters. Indeed, this exercise modality proves to be central for body weight management among overweight and obese adults (Ismail *et al*. 2012).

The idea of which exercise modality could have a greater influence on adiposity‐related anthropometric parameters cannot be elaborated further, because only three investigations comparing different exercise modalities were found. From the obtained data, it seems that aerobic interval training might have certain advantages over continuous aerobic training, while combined endurance and muscular training do not exhibit a greater effect than aerobic exercise alone. This is supported by similar findings from previous works evaluating other populations (Monteiro *et al*. 2015; Wewege *et al*. 2017).

In this piece of research, publications including both adult and young populations mostly with mild to moderate ID were reviewed. Although ID causes were not always reported, many investigations focused on young people with DS were found. This is a fact that deserves some appreciation because this group of individuals presents a high obesity prevalence (O'Shea *et al*. 2018); but yet, based on previous findings (Bertapelli *et al*. 2016) and also to our results, exercise‐based programmes appear to be insufficient to achieve weight or fat loss.

Recent evidence has identified the difficulty of procuring an effective strategy to reduce obesity among people with ID. For instance, Harris et al. (2018) reported that current multicomponent weight management interventions were not more effective than no treatment in this population. The authors suggested that this lack of effect was due to the interventions did not adhere to clinical recommendations regarding diet, exercise and behaviour change techniques. However, they did show that multicomponent interventions that specifically included an energy‐deficient diet were effective. Similarly, Ptomey et al. (2018) demonstrated that a well‐controlled and designed intervention combining diet, PA and counselling led to significant weight changes in obese people with ID. Altogether, these results suggest that multicomponent interventions are a more effective approach than prescribing exercise alone for obesity management in people with ID.

The main strength of this review lies in its ability to build upon currently existing revisions and meta‐analysis of the RCTs regarding the impacts of exercise on anthropometric and body composition parameters in people with ID, in order to reach more robust conclusions. Nevertheless, some methodological weaknesses must be recognised. First, various reviewed papers did not inform the aetiology and severity of ID. Also, the low number of studies included in the meta‐analyses did not allow a stratified analysis by ID aetiology. Second, most of the studies had samples wholly made up of DS participants. Although this finding was somehow expected, because people with DS are one of the most at‐risk groups within the ID population for weight gain and obesity, it shows the need for further studies including different diagnoses within the ID population. Third, the different procedures to measure adiposity across the included studies limit the quality of the meta‐analysis. Fourthly, investigations with two exercise‐based programmes were not included in the meta‐analysis. Hence, proper advice about the benefits of a specific exercise modality compared with other exercise options could not be given. Fifthly, because of the reduced number of studies included per meta‐analysis, a moderator analysis using meta‐regression could not be performed, which would reduce the risk of type I error (Gordon *et al*. 2018). Sixthly, the analysed evidence might be incomplete because of language restrictions and because the grey literature was not reviewed. Finally, another limitation is the potential selective publication in the scientific literature (publication bias).

## Conclusions

While physical exercise can contribute to adiposity‐related anthropometric parameters in people with mild and moderate ID, these findings show that exercise alone is not sufficient to manage obesity in this population. Multicomponent interventions appear to be the best choice when they incorporate dietary deficit, PA increase and behaviour change strategies. Finding the most effective modality of physical exercise can only aid weight loss interventions. There is a need for future research aimed at comparing the impacts of different exercise modalities within the framework of a multicomponent intervention.

## Source of funding

The authors declare no funding for this research.

## Conflict of interest

The authors declare that there is no conflict of interest.

## Supporting information




**Figure S1.** Forest plot of the meta‐analysis for effects of exercise overall on adiposity‐related anthropometric variables. *CG* control group; *CI* confidence interval; *EG* experimental group; *SMD*, standardised mean difference.
**Figure S2.** Forest plot of the meta‐analysis for effects of exercise overall stratified by age group on adiposity‐related anthropometric variables. *CG* control group; *CI* confidence interval; *IG* intervention group, *SMD* standardised mean difference.

## Data Availability

The authors confirm that the data supporting the findings of this study are available within the article and its supporting information. Raw data that support the findings of this study are available from the corresponding author upon reasonable request.
