# Supplementary material for: Effects of exercise training on obesity‐related parameters in people with intellectual disabilities: systematic review and meta‐analysis
Source: J Intellect Disabil Res. 2022 Mar 16;66(5):413–41. doi: 10.1111/jir.12928 (PMC9314046; doi:10.1111/jir.12928)
Supplement: Supplementary file 1 — Figure S1. Forest plot of the meta‐analysis for effects of exercise overall on adiposity‐related anthropometric variables. CG control group; CI confidence interval; EG experimental group; SMD, standardised mean difference. Figure S2. Forest plot of the meta‐analysis for effects of exercise overall stratified by age group on adiposity‐related anthropometric variables. CG control group; CI confidence interval; IG intervention group, SMD standardised mean difference. [file JIR-66-413-s001.docx]

**Supplementary file 1** Forest plot of the meta-analysis for effects of exercise overall on adiposity-related anthropometric variables. *CG* control group; *CI* confidence interval; *EG* experimental group; *SMD*, standardised mean difference.

**Supplementary file 2** Forest plot of the meta-analysis for effects of exercise overall stratified by age group on adiposity-related anthropometric variables. *CG* control group; *CI* confidence interval; *IG* intervention group, *SMD* standardised mean difference.
